# Posters

**DOI:** 10.1038/sj.bjc.6601937

**Published:** 2004-06-22


### A COMPARISON OF TWO QUALITY OF LIFE INSTRUMENTS IN PATIENTS WITH UPPER GASTROINTESTINAL CANCER

SA Goodbrand [1], J Thomson [2], L Bartelemes [1], PA Levack [2], AM Thompson [1]
[1] Department of Surgery and Molecular Oncology, University of Dundee, Dundee, United Kingdom, [2] Macmillan Cancer Team, Dundee, United Kingdom

Quality of life (QoL) is of particular importance in upper gastrointestinal cancer where only a third of patients have operable disease and palliation may be difficult. The aim of this study was to compare two QoL instruments EORTC QLQ-C30 and SEIQoL-dw, a patient centred questionnaire, in patients with oesophageal, gastric or pancreatic cancer.
Consecutive patients with oesophageal (n=33), gastric (n=18) or pancreatic (n=11) cancer completed both the EORTC and SEIQoL-dw questionnaires at diagnosis and for 20 patients with oesophageal or gastric cancer 6-weeks into treatment for palliation of disease.
The male: female ratio was 5.3: 1 and mean age 73 years (range 55-85). There was significant correlation between the two QoL tools (Pearson correlation 0.82, P<0.01), although this reflected a correlation in emotional functioning (Pearson correlation 0.3, P<0.05) but not in physical functioning. EORTC tool gave lower mean QoL scores than SEIQoL-dw (table 1) but for both tools, the QoL declined over the 6 week period.

| Table 1: | Oesophageal cancer | | Gastric cancer | |
|---|---|---|---|---|
| Before | After | Before | After | |
| | (n=33) | (n=12) | (n=18) | (n=8) |
| EORT | 53.3 | 21.3 | 55.1 | 28.4 |
| SEIQoL-dw | 69.7 | 52.6 | 73.4 | 50.0 |

Although there were correlations between the two QoL tools, different domains were important using the two questionnaires, with family and home issues rated above health in SEIQoL-dw where the patient chooses the 5 most important domains.

Given the multi-dimensional nature of QoL, future studies of QoL in patients with upper gastrointestinal cancer should include patient-centred tools such as SEIQoL.

### P62

### SAFETY AND EFFICACY OF USE OF BONANNO SUPRAPUBIC CATHETER TO DRAIN MALIGNANT PLEURAL EFFUSION

M Moe , A Hussain, Singleton Hospital, Swansea, United Kingdom

Objectives: Despite the availability of licensed small-bore chest tubes (eg. Seldinger portex system) Bonanno suprapubic urinary catheter has been used in UK hospitals. The Bonanno catheter has smaller diameter (2.1 mm) than proper chest tubes (2.33 to 4.67 mm) ever studied. This study evaluates the safety and efficacy of use of Bonanno catheter to drain malignant pleural effusions, and use of different systems in other UK hospitals.
Study design: Retrospective study on pleurocenteses done with Bonanno catheter in one oncology department, and UK postal questionnaire survey regarding use of different systems and Bonanno catheter in other hospitals (Survey was conducted in August 2001.).
Findings: 134 pleurocenteses in 80 cancer patients were evaluated. Total drainage = 279.15 litres for 127 procedures (data not available for 7 procedures); range = 0 – 10 litres; mean = 2.25 litres. Complications included local pain (10.4%); pneumothorax (4.5%); blockage and falling out (1.5% each); broken tube requiring surgical removal (0.7%). After pleurodesis with Bleomycin ( 17 patients) and Tetracycline (19 patients) twelve patients in each group did not require any further drainage in next 30 days. 70 out of 118 hospitals replied questionnaires (62% response rate). Use of different systems were Bonanno catheter: 14 (20%) hospitals; conventional standard tube = 58 (83%) hospitals; Pigtail catheter under ultrasound guidance = 34 (48%) hospitals (it was the sole method used in 3 hospitals); Seldinger portex system = 12 (17%) hospitals. 42 (60%) of hospitals didn't have any knowledge of use of Bonanno catheter for chest drainage. Six hospitals reported experiencing tube blockage, kink, splitting, fracture, slow drainage and pneumothorax with Bonanno catheter.
Conclusions: Bonanno catheter is effective and relatively safe. Tube fracture and kink are the main problems. Similar system with blunt-tip trocar needle, flexible and kink-resistant catheter will be an ideal to drain malignant pleural effusion.

### P63

### WEEKLY CISPLATIN AND ORAL ETOPOSIDE FOR PLATINUM RESISTANT OVARIAN CANCER

S Clive [1], DJ Storey [1], C Cullen [2], N Dixon [2], Rye T [2], M MacKean [1], JF Smyth [2], H Gabra [3]
[1] Edinburgh Cancer Centre, Edinburgh, United Kingdom, [2] Cancer Research UK, Edinburgh, United Kingdom, [3] Imperial College, London, United Kingdom

Studies have suggested high response rates in platinum resistant ovarian cancer using a regime of weekly cisplatin and daily oral etoposide. We report our experience of this regime in a non-selected group of platinum resistant patients.
Patients (pts) with progressive epithelial ovarian cancer who were resistant to platinum were offered weekly cisplatin $60mg/m^2$ for 6/8 weeks and daily oral etoposide 50mg for 4/8 wks (induction) followed by oral etoposide 50mg for up to 9 mths (maintenance). Toxicity, quality of life (QOL) and response data were prospectively collected.
21 pts (med age 59(44-68), PS 1(0-2), med 2(1-3) previous chemotherapy regimes) were treated. 14 (67%) were platinum refractory (PD <4mths post platinum), 6 (28%) platinum resistant (PD <6mths post platinum and 1 had PD 8 mths post platinum, with med platinum free interval 5(0-11) mths. The 1st 5 pts had cisplatin in N saline and 3/5 did not complete induction because of toxicity. 16 received cisplatin in hypertonic saline, enabling higher dose intensity. 7/16 did not complete induction after a med 3 wks cisplatin (3 PD, 3 toxicity). Induction caused delays of med 2(0-4) wks for toxicity (2 patients had no delays). Incidence of grade 3/4 toxicities during induction was: neutrophils 47%, platelets 18%, anaemia 9%, neurotox 3%, vomiting 3%. 3 pts had neutropenic sepsis of whom 1 died. 11 pts started maintenance. 1/11 had 9 cycles of etoposide, 4 continue after med 4 cycles, 6 had med 3(2-7) cycles. There was 1 CR (maintained >28 wks), 3 PR, 4 SD (2/4 with >75% CA125 response), 12 NE (4/12 with >75% CA125 response) and 1 PD giving a total scan response of 19% but total scan and/or CA125 response of 48% for a median of 16 (6-28 wks). Full analysis of QOL is awaited but global QOL did not appear to change significantly between cycles 1 and 2.
Wkly cisplatin/oral etoposide is active in platinum resistant ovarian cancer. However in heavily pre-treated, progressing pts, it is associated with significant neutropenia and should be used with caution.

### P64

### GENETIC ANTICIPATION IN FAMILIAL PANCREATIC CANCER

CD McFaul [1], R Kress [2], DK Bartsch [2], H Rieder [2], M Sina-Frey [2], JP Neoptolemos [1], W Greenhalf [1]
[1] The University of Liverpool, Liverpool, United Kingdom,
[2] Phillips-University, Marburg, Germany

An estimated 10% of pancreatic cancers are inherited. Familial Pancreatic Cancer (FPC) is an autosomal dominant disease. Genetic anticipation refers to the earlier age of onset of familial diseases in successive generations. EUROPAC (the European Registry of Hereditary Pancreatitis and Familial Pancreatic Cancer) and FaPACa (German National Case Collection) have combined their databases in order to investigate the reports of anticipation in FPC. This has given us clues to the nature of the disease gene and is vital for linkage analysis. The database has 115 families with pancreatic cancer, 104 from EUROPAC and 11 from FaPaCa. Individuals were assigned to one of three "Hsu" generations (G1, G2 and G3) depending on their position within the family tree (as described by Hsu et al (2000) Genet Epidemiol, 18, 17-32). Comparison of the age between pairings of affected parents and children indicates that children die from pancreatic cancer 10 years earlier than their parents. This is statistically significant but potentially represents bias. To allow for unaffected individuals we carried out Kaplan-Meier analysis comparing the three "Hsu" generations (logrank p<0.01). This remained statistically significant even when limiting observation time to an equal (60 year) period, allowing consideration of individuals born up to the year 1943 (p<0.01).
If there is no generation effect on the age of pancreatic cancer we would predict that the age related risk from one generation could be used to accurately predict the number of pancreatic cancer cases seen in any other generation (regardless of the period of observation). This was found not to be the case and prediction of cancer required a progressively increasing cancer risk per generation (anticipation). Therefore, anticipation does NOT appear to be a statistical anomaly and we present arguments that anticipation is genetic rather than environmental.

© 2004 Cancer Research UK

### P65

### THE CAREGIVER DISTRESS SCALE, A USEFUL TOOL TO ASSESS THE NEEDS OF CARERS

EM Rankin , E Wilkie , R Earl
United Kingdom, Division of Cancer Medicine, University of Dundee, Dundee

There is evidence of significant distress in the carers of patients with cancer (reviewed Eur.J Cancer 2003 39 1517). Our work and that of others has shown that the carers' requirements are frequently unmet, and that sometimes patients block access of carers to sources of support and information (Eur J Cancer Care 2002 11 183). A simple tool to direct intervention to help the carer in the routine clinical setting is required.
Cousins et al (Br J Clin Psychol 2002 41 387) developed a brief 17 point scale to assess distress – the Caregiver Distress Scale (CDS) using carers of patients with Parkinsons' disease. In a preliminary study to test the utility of the tool, we have used this scale so far in 37 consecutive carers who also completed the Beck Depression inventory and Spielberger State Trait Anxiety Inventory. Patients separately filled in the CDS as if they were answering for their carer.
Carers found the tool easy to use. The CDS was completed by 92% of pairs indicating high acceptability. The results showed considerable underestimate by the patient of the distress experienced by the carer in areas concerning personal cost (Spearman's rank correlation 0.381, p0.046) care-receiver demands (Spearman's $r$ 0.64, p0.013), social impact (Spearman's $r$ 0.485, p0.009) and emotional burden (Spearman's $r$ 0.670, $p<0.001$). There was a significant correlation between depression in the carer and the perceived emotional burden ($p=0.002$) and personal cost ($p=0.024$) of the carer. Trait anxiety correlated with perceived social cost of their role to the carer ($p=0.019$).
The CDS is a useful, easily administered tool which takes 5 minutes to complete. Analysis of the carer's score is rapid, enabling the health professional to target attention to areas of distress. This should lead to better outcomes and improve the carer's level of satisfaction with their role.

### P66

### MULTI-MODALITY TREATMENT IN PRIMARY BONE LYMPHOMA – A SINGLE INSTITUTION'S EXPERIENCE

D Ford [1], D Wilson [1], S Sothi [2], R Grimer [3], D Spooner [1]
[1] United Kingdom, University Hospital Birmingham, Queen Elizabeth Medical Centre, Birmingham, [2] United Kingdom, Walsgrave NHS Trust, Coventry, [3] United Kingdom, Royal Orthopaedic Hospital, Birmingham

*Background*: Primary bone lymphoma (PBL) is a rare disease, with limited specific published data concerning optimal management. We describe the experience of a tertiary referral centre in treating PBL with multi-modality therapy (chemotherapy, radiotherapy and surgery).
*Methods*: Patients with a diagnosis of PBL were identified using the hospital data base. Data pertaining to diagnosis, stage, treatment and outcome were collected retrospectively.
*Patient Characteristics*: Between 1985 and 2003, 22 patients (10 M; 12 F), median age 50 years (range 18-89), with histologically confirmed PBL were treated; 19 had high grade histology, 2 low grade, 1 not graded.17 had stage IE disease and 5 stage IV. International prognostic indices (IPI) were 13 low risk, 8 intermediate and 1 high risk.
*Treatment:* All patients received chemotherapy (1 chlorambucil/ vincristine for low grade disease, 2 MCOP, 13 CHOP, 5 CHOP-Methotrexate, 1 CAPOMET). Additional local treatment was given to 21 patients; 18 received radiotherapy (30-50Gy in 10-25 fraction), median $BED_{10}$ of 48Gy, ($\alpha/\beta$ ratio 10 Gy, range 39-60 Gy). 3 patients had surgery alone as local treatment (2 endoprosthetic replacement (EPR) and 1 fibulectomy).
*Results:* Median follow-up is 84.5 months (range 2-206). 4 patients have died, 3 with disease at the time of death. 18 patients remain alive and disease free; 1 patient has required second line chemotherapy and remains disease-free at 45 months, and 1 patient has developed a second malignancy (Hodgkin's disease). Actuarial 10 year survival is 85% for low risk IPI and 66% for intermediate IPI. 10 year relapse free survival is 88% for low risk and 71% for intermediate risk IPI. Complications included 1 patient with avascular necrosis and 1 patient with a pathological fracture after biopsy.
*Conclusion:* Systemic chemotherapy, followed by local therapy with radiotherapy or surgery, produces excellent results for PBL.

### P67

### PRIMARY B-CELL LYMPHOMA OF THE SKIN

L El-Helw , BW Hancock , D Stater
YCR Academic Unit of Clinical Oncology, Weston Park Hospital, Sheffield, United Kingdom

The clinical presentation, treatment and outcome were retrospectively evaluated in a series of 66 patients with primary B-cell lymphoma of the skin, referred to our centre between 1984 and 2003.
The lymphoma database was searched and clinical records were reviewed. Absence of any detectable extracutaneous lesion and the expression of B cell restricted antigens by neoplastic cells were the essential criteria for selection of cases.
The cohort included 37 (56%) males and 29 (44%) females with a mean age of 59 years. The most commonly involved site was the trunk and the disorder typically showed non aggressive clinical behaviour; the majority of the patients presented with stage I (82%) and/or low grade (50%) disease with a tendency to remain localised to a limited area of the skin. Follicular lymphoma was the most common histologic subtype (35%), the next most frequent was the diffuse large cell lymphoma (32%). The majority (47%) of patients were treated with radiotherapy for localised disease whereas chemotherapy was given in 20% of patients, with single agent chlorambucil being most frequently used. Surgical excision as the sole modality of treatment was adequate in 33%.
Disease free survival (DFS) was 91% at 1 year, 82% at 2 years and 60% at 5 years. DFS was significantly lower with older age (> 45 years), leg lesions, generalised and multiple lesions, and for those treated with chemotherapy. The survival at 5 and 10 years was 80%. The histologic grade, leg involvement and the number of lesions were the most significant variables affecting overall survival. Only 7 patients died of lymphoma.
In conclusion primary cutaneous B cell lymphoma represents a peculiar and relatively homogenous entity concerning clinical behaviour, response to treatment, and overall prognosis. It is a condition with an excellent survival rate compared to other extranodal non-Hodgkin's lymphomas. Treatment should be tailored to each individual patient according to number and distribution of the lesions, histology, and general condition of the patient.

### P68

### AN EVALUATION OF A TEACHING SESSION FOR JUNIOR DOCTORS CONDUCTING DO NOT ATTEMPT RESUSCITATION DISCUSSIONS WITH TERMINAL CANCER PATIENTS

DW Fyfe [1], B El Khoury [2], V Crosby [3], G Walker [2]
[1] Nottingham City Hospital, Nottingham, United Kingdom, [2] Queens Medical Centre, Nottingham, United Kingdom, [3] Hayward House Hospice, Nottingham, United Kingdom

Objective
To evaluate whether communication skills training in discussing "Do Not Attempt Resuscitation" (DNAR) decisions with patients with terminal cancer is perceived to be needed by and of benefit to junior doctors.
Design
A single short training session covering communication skills in approaching and conducting DNAR discussions, with evaluation by questionnaires before and approximately 2 months after.
Setting
Junior doctors' teaching programme at two Teaching Hospitals.
Participants
Senior and Junior (Pre-registration) House Officers from both medical and surgical specialities.
Results
55 junior doctors participated in 5 teaching sessions: 29 senior and 26 junior house officers. 78% had conducted a DNAR discussion in the previous 3 months (median 3 discussions each). 43% had received some form of previous DNAR teaching. All felt they could improve on at least some aspects of discussing DNAR. 85% scored the session as relevant or very relevant. The follow-up questionnaire response rate was 62%: 79% of responding doctors felt that the training session had changed their practice; confidence levels had increased in 65%; their median confidence score in discussing DNAR had improved from 3 to 4 (on a scale of 0-5).
Conclusion
A single teaching session on how to discuss DNAR with terminal cancer patients was found to have a positive subjective impact on practice. Teaching communication skills in how to conduct DNAR discussions should form an integral part of junior doctor training.

© 2004 Cancer Research UK 

### P69

### DETECTION OF HUMAN PAPILLOMAVIRUS DNA TYPES 16 AND 18 IN CERVICAL ADENOCARCINOMA AND ITS PRECURSORS BY PCR

Magdy El-Mansi , ARW Williams , RG Morris,
Edinburgh Medical School, Edinburgh

*Objective*: To determine the prevalence of human papillomavirus (HPV) types16 and 18 in cervical adenocarcinoma and its precursors of Scottish patients. *Methods*: HPV DNA was extracted from paraffin-embedded, formalin-fixed tissues of 161 specimens of 139 patients, including 119 cases of invasive adenocarcinoma and 20 of pre-invasive precursors (high grade CGIN). HPV DNA was detected by PCR test using type specific primers from the *E6* gene and *E7* gene of HPV type 16 and HPV type 18 followed by restriction enzyme digestion. *Results*: Out of a total of 139 women with various cervical adenocarcinomas lesions, HPV DNA was identified in 87 cases (62.6%) in which, HPV16 was negative for 74 (53%) and positive for 65 (47%) patients and HPV18 was negative for 98 (71%) and positive for 41 (29%) patients. Genotyping by RFLP and PCR revealed that HPV type 16 was the most frequent type of HPV detected, comprising 46 cases (33%), followed by HPV type 18 in 22 cases (16%), and HPV type16 and HPV type 18 in 19 cases (14%). There were 52 (38%) of 139 of patients with various cervical adenocarcinoma lesions with HPV type16 and HPV type 18 both negative. HPV typing in all cases of 16 normal cervical biopsies was negative with both HPV type 16 and HPV type 18. *Conclusions:* Our findings support that HPV16 (mainly), along with HPV18, may play a role in pathogenesis of cervical adenocarcinoma and its precursors.

### P70

### EPIRUBICIN-CARBOPLATIN-CAPECITABINE (ECARBOX) IN RELAPSED OVARIAN CANCER: A PHASE I/II TRIAL

R Hubner , T Ahmad , A Rigg , S Kaye , I Gibbens , C Keyzor , A Prouse , M Gore
Royal Marsden Hospital NHS Trust, London, United Kingdom

**Background:** We have previously shown the combination of epirubicin, cisplatin, and prolonged infusional 5-fluorouracil (5-FU) is active in relapsed epithelial ovarian cancer (EOC). Capecitabine (Xeloda, X) is an orally bioavailable tumour selective fluoropyrimidine, it has proven efficacy and is more convenient than infusional 5-FU. We have performed a phase I/II study of ECarboX in relapsed EOC.
**Method:** Patients (pts) with relapsed EOC with an interval of at least 6 months from platinum-based first line treatment, received bolus iv epirubicin 50mg/m$^2$ and iv carboplatin AUC 5 on day 1, and capecitabine either 750mg/m$^2$ (level 1) or 1000mg/m$^2$ (level 2) daily in two divided doses throughout a 21-day cycle, or 1000mg/m$^2$ daily for days 1-14 of a 21-day cycle (level 3). Pts were planned to receive 6 cycles. The primary endpoint was the maximum tolerated dose (MTD).
**Results:** At dose level 1 two pts completed 6 cycles and one pt 4 cycles of treatment. Seven cycles were delayed, and 50% dose reductions in epirubicin and capecitabine were required in two pts: one pt had recurrent grade 3 neutropenia, the other had grade 3 neutropenia, lethargy and diarrhoea, and was subsequently withdrawn for prolonged (>2 week) grade 2 neutropenia despite dose reduction. At level 2, four out of five pts completed 6 cycles, the fifth received 2 cycles. There were 11 treatment delays: 2 pts had 25% dose reductions and 1 pt was withdrawn from the study due to prolonged grade 3 neutropenia. Four pts experienced grade ≥2 haematological toxicity and one pt had grade 3 lethargy. At level 3, three pts have received at least 3 cycles each, and a further 3 pts at least 1 cycle. There have been 4 treatment delays and two 25% dose reductions, both for grade 2 neutropenia. Eleven pts are evaluable for response: 8 pts have obtained a partial or complete response, 1 pt has stable disease, and only 2 pts have progressed.
**Conclusions:** The MTD was level 2, capecitabine 1000mg/m$^2$ for 21 days of a 21-day cycle. Capecitabine on days 1-14 of a 21-day cycle is likely to be better tolerated and is being further explored.

### P71

### MEAT, COOKING METHODS AND RISK OF COLORECTAL CANCER: A CASE-CONTROL STUDY

L. Yaghjyan
American University of Armenia, Yerevan, Armenia

Objectives: The study aimed to explore an association between meat consumption, its cooking methods and risk of colorectal cancer.
Study Methods/Design: The study utilized a case-control design. Seventy-seven patients diagnosed with colorectal cancer during the period from August 17, 2002 to August 20, 2003 were included in the study as cases. The control group was selected from healthy hospital visitors, who were free of the disease, and were not related to the patient. The controls were matched with the cases by age and gender. Information was collected using telephone or face-to-face interviews by means of interviewer-administered questionnaires.
Results: The analysis showed that the risk of having colorectal cancer increased with everyday meat use compared with not-daily meat use (adjusted for frequency of fried and boiled sausage use and preference of fried meat surface: OR=3.2; 95% CI 1.0- 18.5; p-value 0.044), with preference of heavily browned surface of fried meat compared with lightly browned (adjusted for daily meat use and frequency of fried and boiled sausage use: OR= 15.4; 95% CI 2.8-85.8; p-value 0.002). There was no statistically significant risk of having colorectal cancer across different types of meat as well as across preferred cooking methods for different meat types. The results of the study have also shown a protective effect of frequent use (more than once/week) of boiled and fried sausage use on risk of colorectal cancer (adjusted for daily meat use and preference of fried meat surface: OR=0.03; 95% CI 0.004-0.3; p-value 0.002, and OR=0.1; 95% CI 0.008-0.5; p-value 0.008, respectively).
Conclusions: Risk of colorectal cancer increased with everyday meat use and preference of heavily browned meat surface, and decreased with frequent use of boiled/fried sausages.

### P72

### DETERMINATION OF GALECTIN 3 LEVELS IN THYROID TUMOURS BY REAL-TIME PCR AND LOCALISATION BY IMMUNOCYTOCHEMISTRY.

NG Powell [1], LA Stephens [1], S Jeremiah [1], RM Tuttle [2], TI Bogdanova [3], MD Tronko [3], DW Williams [1], GA Thomas [1]
[1] Human Cancer Studies Group, Swansea University, Swansea, United Kingdom, [2] MSKCC, New York, United States, [3] Institute of Endocrinology and Metabolism, Kiev, Ukraine

Galectin-3 (Gal-3) is a beta-galactoside-binding protein implicated in a number of functions, including cell proliferation and differentiation, cell adhesion, angiogenesis, apoptosis, and cell survival. Alterations in the levels of Gal-3 mRNA and protein and in its intracellular localisation suggest that it plays important role in tumourigenesis in a variety of tissues. There have been few direct comparisons of mRNA levels with protein localisation. We compared gal-3 mRNA levels by quantitative PCR (QPCR) and protein localisation by immunocytochemistry (ICC) in paired samples of tumour and normal tissue from 50 cases of papillary carcinoma (PTC) and 14 cases of cellular follicular adenoma (FA) from Ukraine. PTCs showed elevated levels of Gal-3 mRNA on QPCR relative to normal tissue (Wilcoxon test $p< 1.209 \times 10^{-6}$), and predominantly universally positive staining in the cytoplasm of the follicular cells comprising the tumour. Nuclear positivity was observed in nearly all PTCs, but this was not marked. Gal-3 ICC showed positive staining only in endothelial cells in the normal thyroid tissue, whether derived from patients with PTC or FA. Endothelial staining was not seen within PTCs. FAs, in contrast to PTCs showed a lower level of gal-3 RNA on QPCR compared to paired normal tissue (Wilcoxon test $p< 3.209 \times 10^{-5}$). On ICC, only two of the adenomas showed localisation of gal-3 to the follicular cells; the majority showed negative follicular epithelium, but marked positivity of endothelial cytoplasm within the tumour. In two FAs, areas of tumour with nuclear and cytoplasmic changes similar to that seen in PTCs showed strong cytoplasmic positivity for gal-3 protein.
These results suggest that gal-3 may be a useful marker for distinguishing PTC from follicular tumours, but not a suitable for tool in preoperative diagnosis for determining malignancy in follicular tumours. The differences in endothelial positivity between PTCs and FAs are intriguing and warrant further investigation.

© 2004 Cancer Research UK

### P72:1

**CARBOPLATIN + DOXORUBICIN (CD) AND CARBOPLATIN + PACLITAXEL (CP) GIVEN AS A SEQUENTIAL DOUBLET IN ENDOMETRIAL CANCER (EC) AND MIXED MESODERMAL TUMOURS (MMT).**

RNH Shah , M Everard , C Keyzor , K Hall , R Ahern , ME Gore , SB Kaye
The Royal Marsden Hospital, Sutton, United Kingdom

Platinum, anthracycline and taxane based regimens are active in advanced/recurrent EC and MMT. However, the toxicity of concurrent 3-drug regimens may preclude their routine use in a palliative setting. We are performing a feasibility study using a sequential doublet consisting of 4 cycles of CD (C=AUC5, D=50mg/m$^2$, d1q21) followed by 4 cycles of CP (C=AUC5, P=175mg/m$^2$, d1q21) in patients (pts) with advanced/recurrent Stage III/IV EC, MMT and gynaecological malignancy where the histological differential lies between endometrial and ovarian cancer. We hypothesise that this would allow all 3 drugs to be given with acceptable tolerability. 19 pts have enrolled, of whom 15 have completed all planned therapy. Diagnosis is EC in 14 pts and MMT in 5 pts. Median age is 56.
Pts have received 126 cycles of chemotherapy of a planned maximum of 134 (94%). Of these 126 cycles, 23 were delayed by 1 week and 3 by 2 weeks. Reasons for delay were exclusively haematological (73% neutropenia, 27% thrombocytopenia). Dose reductions occurred 5 times and were continued for subsequent cycles in any given patient. Causes for dose modification were myalgia (1pt), parasthesia (1pt) and neutropenia (3pts). Non-haematological toxicity was as expected and included universal alopecia. Grade II toxicity consisted of nausea (2pts), vomiting (2pts), constipation (1pt), myalgia (1pt) and sepsis (1pt). Grade III toxicity consisted of myalgia (1pt) and parasthesia (1pt). No grade IV non-haematological toxicity was seen.
Response data in 11 pts with measurable/evaluable disease shows CR in 3 pts, PR in 6 pts and PD in 2 pts. Disease was non measurable throughout in 5 pts. 2 pts have died to date, one of PD after 4 cycles and another after 7 cycles having initially responded.
Our data suggest this sequential doublet is a feasible regimen, combining tolerable toxicity with impressive response data, and warrants further investigation.

### P72:2

**MOTIVATING SMOKING CESSATION TO REDUCE THE RISK OF CERVICAL CANCER**

S Hall [1], TM Marteau [1], AJ Bishop [1], H Kitchener [2], P Hajek [3]
[1] United Kingdom, Institute of Psychiatry, Kings College London, London, [2] United Kingdom, University of Manchester, St. Mary's Hospital, Manchester, [3] United Kingdom, Queen Mary's School Of Medicine and Dentistry, Barts and The London Hospitals, London

**ABSTRACT WITHDRAWN**

### P72:3

**SPACE-TIME CLUSTERING ANALYSES OF CHILDHOOD CANCERS SUPPORTS A COMMON INFECTIOUS AETIOLOGY**

RJQ McNally [1], OB Eden [2], FE Alexander [3], AM Kelsey [2], JM Birch [1]
[1] United Kingdom, Cancer Research UK Paediatric and Familial Cancer Research Group, Manchester, [2] United Kingdom, Central Manchester and Manchester Children's University Hospitals NHS Trust, Manchester, [3] United Kingdom, University of Edinburgh, Edinburgh

In previous studies we demonstrated significant space-time clustering amongst cases of ALL, astrocytoma, soft tissue sarcoma and Wilms' tumour. We hypothesised that there may be a common aetiology particularly between some of these diagnostic groups. The aim of the present study was to test this hypothesis by analysing for cross-clustering between cases in different diagnostic groups. Cases included in a population-based childhood cancer registry during the period 1954-2001 were analysed. Knox tests for space-time interactions between cases were applied with fixed thresholds of close in space, <5km and close in time, <1 year apart, to determine whether there are more pairs occurring in close proximity than expected by chance. Tests were repeated replacing geographical distance with distance to the N$^{th}$ nearest neighbour [NN] to adjust for population density. N was chosen such that the mean distance was 5km. Data were also examined by a second order procedure based on K-functions to allow for multiple testing and boundary effects. Reference points in time and space were dates and addresses at birth and diagnosis respectively.
All four methods showed statistically significant ($p<0.05$) cross-clustering between cases of HD and astrocytoma, ALL and astrocytoma, and ALL and NHL, based on time and place of birth; between cases of NHL and PNET's, and AML and peripheral neuroectodermal tumours, based on time and place of diagnosis; between cases of ALL and PNET's, and ALL and peripheral neuroectodermal tumours, based on time of diagnosis and place of birth; between cases of ALL and peripheral neuroectodermal tumours based on time of birth and place of diagnosis. There was little evidence of cross-clustering between Wilms' tumours, soft tissue sarcomas and other malignancies respectively.
These findings are consistent with a common infectious aetiology for certain haematological and neural malignancies in children.

### P72:4

**PHARMACOKINETIC MEASUREMENTS OF A 5FU PRO-DRUG, CAPECITABINE, IN BLADDER TUMOURS OVER-EXPRESSING THYMIDINE PHOSPHORYLASE**

Y-L Chung [1], H Troy [1], IR Judson [2], R Leek [3], MO Leach [2], M Stubbs [1], AL Harris [3], JR Griffiths [1]
[1] United Kingdom, St George's Hospital Medical School, London, [2] United Kingdom, Institute of Cancer Research, Surrey,
[3] United Kingdom, John Radcliffe Hospital, Oxford

Capecitabine is a prodrug of 5-fluorouracil (5'FU). The final step of conversion is from 5′deoxy-5-fluorouridine (5′DFUR) to 5'FU by thymidine phosphorylase (TP) in tumours. Previous studies have shown that tumour response is strongly correlated with TP levels in tumours. The aims of this study were: a) to investigate the pharmacological roles of TP by measuring the pharmacokinetics (PK) of capecitabine; b) to develop the use of PK measurements of capecitabine by $^{19}$F MRS as a non-invasive surrogate marker for determining TP levels in tumours.
Human bladder xenografts were grown subcutaneously in MF1 nude mice. TP over-expressing (2T10) and control (wild-type (RT112) and empty-vector (EV11)) tumours were examined. When tumours were established (~500mg), mice were given a single dose of capecitabine (360mg/kg in DMSO i.p) or 5'DFUR (200mg/kg in DMSO i.p). $^{19}$F spectra were acquired and the rate constant of capecitabine breakdown, the build-up of the intermediate molecules (5'DFCR/5'DFUR) and the subsequent breakdown of the molecules were determined. The rate constant of the 5'DFUR breakdown was also evaluated.
The rate constant of the breakdown of the intermediate molecules was significantly faster in the 2T10 tumours than in the control group. No significant differences in the rate of capecitabine breakdown or the accumulation of the intermediate molecules were observed. The rate constant of the breakdown of 5'DFUR in the 2T10 tumours was found to be doubled when compared with the controls.
This study confirmed the expected pathway of capecitabine metabolism and it also showed that the level of TP is related to the rate of 5′DFUR conversion. Using *in vivo* $^{19}$F MRS to measure the pharmacokinetics of capecitabine and its intermediate metabolites in tumours may provide a non-invasive surrogate method for determining TP levels in tumours and for predicting tumour response to capecitabine in patients.

© 2004 Cancer Research UK

### P72:5

### DUODENO-OESOPHAGEAL REFLUX ENHANCES MUTAGEN INDUCED OESOPHAGEAL CARCINOGENESIS

Z Bell [1], C Menezes [1], P Bonde [1], C Swarbrick [3], J Sloan [3], S Mirvish [4], J McGuigan [2], FC Campbell [1]
[1] United Kingdom, Surgery, Cancer Centre, The Queen's University of Belfast, Belfast, [2] United Kingdom, Cardio-thoracic surgery, Cancer centre, The Queen's University of Belfast, Belfast, [3] United Kingdom, Pathology, School of Medicine, The Queen's University of Belfast, Belfast, [4] United Kingdom, Eppley Institute for Research in Cancer, University of Nebraska Medical Centre, Omaha

**Introduction**: Epidemiological evidence points to an association between gastro-oesophageal reflux disease and the rising incidence of oesophageal cancer in the western world. Reflux of duodenal content may be particularly implicated in carcinogenesis but its precise role and magnitude of effects are unclear.
**Aim**: The aim of this study was to assess the effect of duodenal reflux on oesophageal tumourigenesis, either alone or in combination with a human dietary carcinogen, methyl-n-amyl-nitrosamine (MNAN).
**Methods**: Reflux was promoted by duodeno-oesophageal anastomosis without gastric bypass, in male Sprague Dawley rats. Effects of reflux alone or in combination with MNAN (IP injection, 25mg/kg/wk x 4 weeks) were assessed upon inflammatory changes, metaplasia, dysplasia and carcinoma. Rats were randomly assigned to 4 groups: control (n=9), MNAN alone (n=39), reflux alone (n=39) and reflux + MNAN (n=50). Animals were sacrificed at 38 weeks.
**Results**: Groups varied significantly (ANOVA) with regard to inflammation(INF), metaplasia (MP), dysplasia (DYS) and carcinoma (CA).

| | Control % | MNAN % | Reflux % | Reflux + MNAN % | p-value |
|---|---|---|---|---|---|
| INF | 0 | 2.6 | 48.7 | 60 | <0.001 |
| MP | 0 | 0 | 30.8 | 24 | <0.01 |
| DYS | 0 | 15.4 | 38.5 | 54 | <0.001 |
| CA | 0 | 2.6 | 2.6 | 16 | <0.05 |

**Conclusions**: Duodeno-oesophageal reflux promotes oesophageal carcinogenesis in a step-wise progression from inflammation to metaplasia, dysplasia and invasive carcinoma. In addition, dietary carcinogen exposure may enhance this risk.

### P73

### THE INFLUENCE OF POSITRON EMISSION TOMOGRAPHY (PET) IN CHANGING TREATMENT OPTIONS IN LOCALLY ADVANCED AND METASTATIC COLORECTAL CANCER (CRC).

C Thomas , Z Kassam , W Wong , M Harrison
Mount Vernon Cancer Centre, London, United Kingdom

Introduction
Increasing treatment options (including the potential for cure) have led to a more complex treatment algorithm in advanced colorectal cancer. More detailed diagnostic and functional imaging is required to offer patients optimal therapy. PET scanning offers additional confirmation or refutation of the presence of metastatic disease whilst also defining the extent of the disease.
Aim
To assess the changes in management following PET scanning in patients with advanced CRC.
Method
All patients with advanced CRC are routinely staged with spiral CT +/- MRI. Additional PET scanning was performed if the original imaging was inconclusive about disease extent. Forty three (43) PET scans were performed, for this reason, over a 24 month period starting 1/1/02.
Patient characteristics: average age 61.9 years (23-79); male: female ratio 1.5:1; Primary site: rectum (27), sigmoid (7), colon (2), caecum (7); Dukes stage: B (10), C (29), unknown (4).
Results
Additional information, above that already available, was provided in 30 patients (70%). Previous staging assessment was confirmed in the remainder. In the 30 patients 20 were upstaged, 9 downstaged and 1 showed a differential response to previous therapy. Management was changed in 22 (51%) of these patients. An economic analysis is currently being undertaken and will be presented.
Conclusion
Within this cohort of patients 22 (51%) had their management significantly changed as a result of PET scanning. This appears to be a practical and cost-effective form of diagnostic imaging for patients with advanced CRC.

### P74

### INFRARED MICROSPECTROSCOPY IN CANCER DIAGNOSIS

MJ Tobin [1], Ma Chesters [1], J Chalmers [1], Se Fisher [2], Fjm Rutten [3], Im Symonds [3], A Hitchcock [3], Ka Maclennan [2], R Allibone [3], S Dias-gunasekara [1]
[1] United Kingdom, Cclrc, Daresbury, [2] United Kingdom, University Of Leeds, Leeds, [3] United Kingdom, University Of Nottingham, Nottingham

Infrared microspectroscopy is capable of detecting subtle biochemical changes within tissues. The brightness of the synchrotron source combined with microscope access brings the potential to examine tissue at single cell level. We have used this approach to study oral epithelial tumour tissue using synchrotron infrared microspectroscopy.
Material for analysis was prepared from excised tumour specimens using fresh frozen, air dried tumour sections at 5µm thickness. Spectra were collected from areas identified visually as tumour and stroma respectively. After correction for water vapour contributions, all spectra were normalised to the peak height of the amide II absorption, which has been used successfully elsewhere. The region of the remaining spectrum was subjected to multivariate analysis using the Pirouette software package (Infometrix, Inc, Woodville, USA).
Mid infrared absorption spectra, collected at cellular spatial resolution gave results which were sufficiently distinct to distinguish between three different cell types (malignant, stromal and keratinised) when analysed using principal component analysis.
The resulting data were robust enough to achieve correct classification of cells from subsequent specimens.
These results confirm the promise of this new and advancing technology as a future tool for cancer diagnosis in that they show the ability of IR microspectroscopy, together with multivariate data analysis to predict subtle chemical differences between cell types within a tumour. The potential for use in tissue diagnosis merits further exploration, especially in view of the technical improvements that are being made in laboratory level detectors and the ability of this technology to aid pathological tumour diagnosis.

### P75

### DETERMINATION OF [$^{18}$F]FLUOROTHYMIDINE KINETICS IN TUMOUR AND NORMAL TISSUES OF PATIENTS WITH BREAST CANCER USING POSITRON EMISSION TOMOGRAPHY .

L Kenny [1], A Al-Nahhas [1], S Shousha [1], S Osman [2], DM Vigushin [1], RC Coombes [1], EO Aboagye [1]
[1] Imperial College London, London, United Kingdom, [2] Hammersmith Imanet, London, United Kingdom

**Aim**: The aim of this study was to model the tissue kinetics of [$^{18}$F]fluorothymidine ([$^{18}$F]FLT ) positron emission tomography (PET) data in order to derive indices of cellular proliferative capacity.
**Background**: [$^{18}$F]FLT is a substrate for mammalian thymidine kinase 1 (TK1) gene product. TK1 expression increase in the G1-phase of the cell cycle making [$^{18}$F]FLT a potential radiotracer for imaging cellular proliferation *in vivo*. Quantitative analysis by mathematical modelling can, thus, provide parameters that reflect proliferative potential.
**Methods**: Eight tumour areas from five patients with locally advanced or metastatic epithelial breast cancer (lobular carcinoma and ductal carcinomas) were analysed in this study. [$^{18}$F]FLT was administered by intravenous injection followed by dynamic imaging for 90 minutes. Arterial blood samples were analysed by γ-counting and high performance liquid chromatography to derive an arterial input function. Data-led spectral and graphical (Patlak) analyses were performed and compared to semi-quantitative uptake data (SUV).
**Results**: [$^{18}$F]FLT kinetics in tumour showed a linear fit with mean ± s.e. $K_i$ values (net irreversible clearance of radiotracer from plasma to tissue) of 4.71±0.71 x $10^{-4}$ (ml plasma/ml of tissue/sec) indicating retention. A kinetic component approximating the decay constant for fluorine-18 was seen in spectral analysis modelling that was consistent with the $K_i$ parameter. Radiotracer delivery ($K_1$) and fractional retention at 90 min compared to 1 min (FRT) were 0.003±0.0005 ml plasma/ml of tissue/sec and 0.14±0.03, respectively. The SUV at 90 min was 7.925±1.0825 $m^2$/ml. $K_i$, SUV, FRT and $K_1$ were higher in a ductal carcinoma with high Ki-67 labelling than in a lobular carcinoma with low labelling index.
**Conclusion**: The kinetics of [$^{18}$F]FLT can be modelled by spectral and graphical methods to yield parameters that may be related to proliferative capacity.

© 2004 Cancer Research UK

### P76

### 3'-DEOXY-3'-[$^{18}$F]FLUOROTHYMIDINE AND 2-[$^{18}$F]FLUORO-2-DEOXY-D-GLUCOSE AS MARKERS OF RESPONSE TO CISPLATIN THERAPY *IN VIVO*

J Leyton , M Perumal , H Dhaliwal , J Litago , E Aboagye
Hammersmith Hospital Campus, London, United Kingdom

Cisplatin is one of the most widely used chemotherapeutic agent in the treatment of cancer, with wide ranging effects on solid tumours. The aim of this study was to establish whether [$^{18}$F]fluorothymidine ([$^{18}$F]FLT) and [$^{18}$F]fluorodeoxyglucose ([$^{18}$F]FDG) could be used as markers to determine early response to cisplatin therapy.

RIF-1 tumour bearing mice were untreated or treated with cisplatin at single i.p. dose of 5 mg/kg. [$^{18}$F]FLT and [$^{18}$F]FDG were injected intravenously before or at 24 and 48 h after drug treatment. The tissue: blood radioactivity ratios(T:B) were determined from biodistribution studies at 60-min post radiotracer injection. The molecular determinants of [$^{18}$F]FLT uptake, thymidine kinase 1 (TK1) protein and ATP levels, were assessed by western blot and bioluminescence, respectively. Proliferating cell nuclear antigen (PCNA) status was determined.

T:B for [$^{18}$F]FLT pre-treatment, 24 h and 48 h post-treatment were 1.35±0.14, 1.13±0.2 and 1.18±0.11, respectively. This was not statistically significant. Corresponding values for [$^{18}$F]FDG were 10.84±4.2, 8.34±1.9 and 6.12±1.4, which was significant (p=0.03) at 48 h. Tumour volume decrease by 55% at 48 h post treatment. The decrease in [$^{18}$F]FDG uptake correlated with PCNA labelling index (r=0.55; p=0.01). Compared to pre-treatment levels, TK1 protein decreased at 24 h (24.4±11.07%, p=0.01), with partial recovery at 48 h (38.3±22.1%). In contrast, a time dependent decrease in ATP levels was observed at 24 and 48 h post-treatment (p=0.05).

From this initial biodistribution study, [$^{18}$F]FDG was a more specific marker of response to cisplatin therapy. Changes in [$^{18}$F]FLT uptake could be explained in part by the changes in TK1 levels. Positron emission tomography studies are ongoing to fully characterise the radiotracer kinetics.

### P76:1

### CORRELATIVE STUDY OF SPIRAL CT FEATURES AND ANGIOGENESIS IN HAPATOCELLULAR CARCINOMA

B Wang [1], ZQ Gao [2], DX Yu [1], YH Shi [1]
[1] Department of Radiology, the Affiliated Hospital, Weifang Medical University, Weifang, China, [2] Department of Medical Biology, Weifang Medical University, Weifang, China

**Introduction** Hepatocellular carcinoma (HCC) is a high vascular supply tumour. The angiogenesis is one of the most important phenomena in the process of HCC happening, and the process of the angiogenesis is also the necessary in the growth and metastasis of the tumour. Our study is to study the correlation between the contrast enhancement features on spiral CT (SCT) and microvessel density (MVD), diameter and expression location of F-VIII RA in HCC, and the relation between SCT features and clinicopathological data.

**Materials and Methods** Fifty cases (54 lesions) of HCC proved pathologically and examined with enhancement dual-phase SCT scanning (the arterial and the portal vein phase) were included. The expressions F-VIII RA were detected with immunohistochemical SP method. The features of HCC in SCT were compared with the immunohistochemical results of F-VIII RA and clinical and histopathological characteristics of HCC.

**Results** Immunohistochemical results showed that the percentage of capillary-like type, sinusoid-like type and mixed type was 24.1% (13/54), 18.5% (10/54) and 57.4% (31/54) respectively. The mean of MVD and microvessel diameter was 50.20 ± 13.89, 15.06 ± 7.76 µm respectively in 54 lesions. There was significantly correlation between MVD and the size, ascites, Edmondson's grade, and between microvessel diameter and AFP value. The factors related with the enhancement style were MVD, the degree of necrosis and invasion, and the key factor related with the pseudocapsula style were Edmondson's grade and microvessel diameter.

**Conclusion** Some biological behavior of HCC may depend on angiogenesis, and the features of spiral CT may reflect angiogenesis in HCC to some degrees.

### P76:2

### TUMOUR DOSE RESPONSE TO 5,6-DIMETHYLXANTHENONE-4-ACETIC ACID (DMXAA) ASSESSED BY *IN VIVO* MAGNETIC RESONANCE SPECTROSCOPY

LD McPhail [1], YL Chung [1], B Madhu [1], S Clark [2], JR Griffiths [1], LR Kelland [2], SP Robinson [1]
[1] United Kingdom, St. George's Hospital Medical School, London,
[2] United Kingdom, Antisoma Research Laboratories, London

DMXAA is a low molecular weight drug that induces the occlusion and collapse of established tumour blood vessels. The aim of this study was to investigate the dose response of murine HT29 xenografts to DMXAA using magnetic resonance spectroscopy (MRS) and identify early, relevant biomarkers associated with tumour response. *In vivo* $^{31}$P and $^{1}$H MRS was used to assess the bioenergetic status of HT29 tumours prior to and 6 hours after treatment with either 0, 7.5, 15, 21 or 25 mg/kg DMXAA. Following *in vivo* MRS tumours were excised and both high resolution MRS and HPLC were performed on tumour extracts. A section of the tumour was also used for histological analysis of necrosis.

The *in vivo* $^{31}$P-MRS data showed a dose-dependent decrease in high-energy phosphates and increase in low energy phosphate treatment. This was associated with a dose-dependent increase in lactate observed using *in vivo* $^{1}$H-MRS. The $^{1}$H-MRS extract data showed a significant increase in free choline and a significant decrease in glycerophosphocholine (GPC). The *in vivo* dose response was further supported by the HPLC studies on tumour extracts. Importantly, there was no apparent correlation of metabolic changes with the degree of necrosis observed by histology.

The decrease in tumour energetics observed using $^{31}$P-MRS indicates that DMXAA is starving the tumour of its energy supply. The increase in free choline (a precursor of membrane phospholipid) and decrease in GPC (an end product of membrane degradation) suggests that DMXAA is causing a reduction in tumour cell proliferation and membrane turn-over. The increase in lactate after drug treatment is consistent with vascular shut-down as pyruvate, the end-product of glycolysis, is reduced to lactate under anaerobic conditions. The lack of correlation between the *in vivo* MRS data and histology suggests that *in vivo* MRS affords an early, non-invasive assessment of tumour response to DMXAA, and therefore may have potential for use in clinical trials.

### P76:3

### SR4554 AS A NON-INVASIVE PROBE OF TUMOUR HYPOXIA DETECTED BY MAGNETIC RESONANCE SPECTROSCOPY: RESULTS OF A PHASE I TRIAL

CP Lee [1], GS Payne [2], RR Ruddle [1], FI Raynaud [1], SK Tan [1], MJ Campbell [3], M Tracy [4], JP McNamara [4], MO Leach [2], IR Judson [1], P Workman [1]
[1] United States, Cancer Research UK Centre For Cancer Therapeutics, The Institute of Cancer Research and The Royal Marsden NHS Trust, Sutton, [2] United Kingdom, Cancer Research UK Clinical Magnetic Resonance Research Group, The Institute of Cancer Research and The Royal Marsden NHS Trust, Sutton, [3] United Kingdom, Drug Development Office, Cancer Research UK, London, [4] United Kingdom, SRI International, Menlo Park, California

**Introduction:** SR4554 is a fluorinated 2-nitroimidazole which is reduced and bound in hypoxic cells. It was designed for use as a non-invasive tumour hypoxia probe detectable by $^{19}$F Magnetic Resonance Spectroscopy (MRS). The earlier part of our Phase I study of SR4554 showed a plasma $T_{1/2}$ of ~3.3 hr, and a maximum tolerated dose of 1400mg/m$^2$. The 2nd part of this study aimed to further investigate evidence of SR4554 retention in tumour at 16 hr (5x $T_{1/2}$ ) using MRS compared with measured plasma values. **Methods:** Eligibility criteria included tumours ≥ 3cm at a depth of ≤ 4cm. $^{19}$F MRS measurements were performed using a 1.5T Siemens Vision MR system and a 5, 10 or 16cm dual resonant $^{1}$H/$^{19}$F surface coil to approximately localise the signal to the tumour. All patients (pts) received SR4554 at 1400mg/m$^2$ (IV) followed immediately by an MRS scan (MRS #1). The 2nd MRS scan (MRS #2) was acquired at ~16 hr post-infusion to detect $^{19}$F signals indicative of tumour hypoxia following washout of parent SR4554 from tumour. The retention index (RI) (%) was defined as (MRS #2/ MRS #1)*100. Pharmacokinetic (PK) studies were performed. **Results:** 16 pts were enrolled. Toxicities: Grade 1 nausea and vomiting (n=2), Grade 1 rash (n=1). PK studies showed: mean $C_{max}$ = 97.6 ± 48.8 mg/L; mean $T_{max}$ = 0.86 ± 0.25 hr; mean $T_{1/2}$=3.8 ± 0.9 hr, mean $V_{ss}$ = 36.5 ± 17.0 L; clearance = 10.5 ± 4.7 L/hr. Unlocalised MRS studies were acquired in 14/16 pts. SR4554 signals were seen in MRS #1 in all 14 pts. In MRS #2, SR4554 signals were above detection threshold in 8 pts, yielding a mean RI of 10.2 ± 6.6% (range 0.6-21.1%) compared with 2.96 ± 1.85 % (range 0.63-7.33%) in plasma. MRS #2 data were below detection threshold in 6 pts. **Conclusions:** SR4554 at 1400mg/m$^2$ was well-tolerated. We have demonstrated that SR4554 is retained in tumours relative to plasma and that the study is clinically feasible. The range of values for RI relative to plasma supports the hypothesis that this may be a genuine indication of tumour hypoxia.

© 2004 Cancer Research UK

### P76:4

### *IN VIVO* SUSCEPTIBILITY CONTRAST MRI OF MURINE MODELS OF LIVER METASTASIS

TL Kalber [1], SP Robinson [1], FA Howe [1], AJ Ryan [2], JC Waterton [2], JR Griffiths [1]
[1] United Kingdom, St. George's Hospital Medical School, London,
[2] United Kingdom, AstraZeneca, Macclesfield

Ultrasmall superparamagnetic iron oxide (USPIO) particles are iron oxide crystals typically coated with a dextran-based coating with an overall particle size of <50nm. They are selectively taken up by the reticuloendothelial system (macrophages and Kupffer cells) in the liver. When placed in a magnetic field, USPIO particles produce large magnetic susceptibility inhomogeneities, resulting in a dramatic and positive enhancement of $R_2$ and $R_2$* relaxation rates and suppression of normal liver MRI signal intensity. This can facilitate the subsequent detection of metastatic lesions, which do not take up USPIO's and hence "shine through" conspicuously on a darkened background of normal liver parenchyma. To establish the duration and magnitude of suppression of normal murine liver signal afforded by a single injection of USPIO particles, normal murine liver $R_2$* and $R_2$ were quantified longitudinally for up to 50 days following administration of 2.5mgFe/kg AMI-7228 (Ferumoxytol, Guerbet Europe). AMI-7228 dramatically increased liver $R_2$* and $R_2$ at day 0. However, by day 10, liver $R_2$* and $R_2$ of mice administered AMI-7228 had recovered to normal (non-USPIO loaded) levels.
For the investigation of metastatic lesions, mice were intrasplenically injected with $2x10^6$ LS174T or SW1222 colorectal cells. Longitudinal $R_2$* and $R_2$-weighted MRI prior to and following adminstration of 2.5mgFe/kg AMI-7228 was then used to follow the development of metastatic deposits within the liver. Metastases were detected as early as 20 days post i.s. injection and their progression, which was clearly evident, monitored at subsequent timepoints up to 38 days. The data demonstrate proof-of-principle of susceptibility-contrast MRI to investigate mouse models of liver metastasis and how this approach will be used to i) increase the understanding of the basic pathophysiology of liver metastasis *in vivo* and ii) demonstrate the utility of the MRI method for the detection of metastatic disease that can potentially be translated onto conventional clinical MRI instruments.

### P76:5

### USE OF INTRINSIC-SUSCEPTIBILITY MAGNETIC RESONANCE IMAGING TO ASSESS ACUTE TUMOR RESPONSE TO THE VASCULAR-TARGETING AGENT ZD6126

SP Robinson [1], TL Kalber [1], FA Howe [1], DJO McIntyre [1], JR Griffiths [1], DC Blakey [2], L Whittaker [2], AJ Ryan [2], JC Waterton [2]
[1] United Kingdom, St. George's Hospital Medical School, London,
[2] United Kingdom, AstraZeneca, Macclesfield

ZD6126 is a vascular-targeting agent that causes the selective destruction of tumor blood vessels, cessation of blood flow and death of tumor cells due to nutrient starvation, resulting in massive necrosis. We have previously shown the antivascular effect of 50mg/kg ZD6126 on rat GH3 prolactinomas to be profound 24 hours after administration.
Intrinsic-susceptibility magnetic resonance imaging (MRI), in which endogenous deoxyhaemoglobin can provide a source of image contrast, was used to assess the response of rat GH3 prolactinomas to ZD6126. Multi gradient echo (MGRE) MRI was used to quantify the effective transverse relaxation rate $R_2$* which, in the absence of other changes, depends on tissue deoxyhaemoglobin levels and hence may provide an acute index of changes in tissue oxygenation. We hypothesised that following treatment with ZD6126, haemoglobin within erythrocytes would deoxygenate, resulting in an increase in tumour $R_2$*.
Tumour $R_2$* was measured prior to and either immediately (at 7, 14, 21, 28 and 35 minutes) following or 24 hours following administration of 50mg/kg ZD6126. A significant <u>increase</u> in tumour $R_2$* was measured as early as 14 minutes, reaching 116 ± 4% of baseline by 35 minutes after challenge, consistent with an ischaemic insult induced by vascular shutdown/collapse. A strong positive correlation between baseline tumour $R_2$* and the subsequent increase in $R_2$* measured 35 minutes after treatment was obtained, suggesting that the baseline $R_2$* can predict for the likelihood and magnitude of subsequent tumour response to ZD6126. In contrast, a highly significant <u>decrease</u> in tumour $R_2$* to 45 ± 4% of baseline was found 24 hours after administration of ZD6126. Both $R_2$* responses were associated with a decrease in tumour perfusion as measured by Hoechst 33342 uptake. Interpretation of the $R_2$* response is complex, yet changes in tumour $R_2$* may provide a convenient and early MRI biomarker for immediate detection of tumour activity of vascular targeting agents.

### P76:6

### LINEAR DISCRIMINANT ANALYSIS CLASSIFICATION OF BRAIN TUMOUR $^1$H MR SPECTRA: A COMPARISON OF COMPLETE SPECTRA VERSUS QUANTIFICATION

KS Opstad , C Ladroue , JR Griffiths , FA Howe
United Kingdom, St. George's Hospital Medical School, London

Pattern recognition analysis of $^1$H MRS brain tumour spectra may provide a clinically valuable diagnostic tool, but distinction of glioblastomas from metastases is currently difficult. In this study we investigate the use of pattern recognition of single voxel STEAM TE 30ms spectra to compare classification using: i) the whole spectra versus LCModel quantification of 14 biochemicals; and ii) principal component analysis (PCA) versus manually chosen biochemical concentrations from LCModel quantification. Classification was evaluated using leave-one-out linear discriminant analysis (LDA)
Histopathologically verified tumours consisted of 7 astrocytoma grade II (AS2), 15 meningiomas (MNG), 14 glioblastomas (GBM) and 10 metastases (MET). Data reduction of whole spectra was performed using PCA. Manual selection of the quantified biochemicals prior to LDA was according to Mann Whitney U-test statistical comparisons between tumour groups, for which we found the Cr, Ins, GSH and 1.3ppm lipid/macromolecule peaks showed the greatest differences between groups. Leave-one-out LDA classification was performed on the three types of reduced dataset for the following groups: **I**) AS2, MNG and HG (high-grades comprising GBM + MET), **II**) AS2, MNG, GBM and MET; and **III**) AS2, GBM and MET.
LDA of Group **I** on either whole spectra or 4 manually selected LCModel concentrations gave similar result of 91% correct classification. LDA of Group **II** resulted in much poorer classification with between 59 - 80% correct. LDA of Group **III** using PCA of the whole spectra or LCModel quantification was also poor with between 54 - 72% correct. In contrast, using the Cr, Ins, GSH and 1.3ppm lipid LCModel concentrations gave 87% correctly classified spectra, and 83% between GBM and MET alone. This suggests that PCA is not always optimal for data reduction of spectra, and using prior knowledge of quantitative metabolite data may give the best classification.

### P76:7

### A 2.05PPM $^1$H MRS MOBILE LIPID CHARACTERISTIC OF NON-NECROTIC METASTASIS

KS Opstad , JR Griffiths , FA Howe
United Kingdom, St George's Hospital Medical School, London

For patients with brain tumours, determination of tumour grade, and differentiation of metastasis (MET) and glioblastoma (GBM), are clinically important questions. Increased lipids are associated with increased malignancy, and the ≈2.02 ppm NAA peak with normal brain tissue. We now report on a ≈2.05ppm resonance from mobile lipids characteristic of some metastases.
Short echo (TE 30ms) $^1$H spectra were acquired at 1.5T from histologically proven METs (n=22) and GBMs (n=20). In 6 MET patients we observed a narrow peak at ≈2.05ppm: for 2 of these we further acquired spectra with TE = 30, 60, 90 and 136ms and for 2 we acquired metabolite nulled spectra (TE 30ms with a pre-inversion pulse).
The 2.05ppm peak was still present at the longest TE, and we determined it to have an apparent $T_2$ of ~140ms. In the metabolite-nulled spectra the 2.05 peak was clearly present, whereas signals from metabolites choline, creatine and lactate were nulled. Difference spectra revealed only metabolites, indicating the 2.05 ppm peak has much shorter $T_1$ than metabolites, and so likely to be mobile lipid.
Our data indicate there is a mobile lipid moiety which gives rise to a narrow peak at ≈2ppm, and has a longer apparent $T_2$ relaxation time than for other lipid/macromolecule peaks ($T_2$ < 70ms) found in brain tumours. This could be mistaken for an NAA signal in long TE spectra, and so cause an underestimation of malignancy. We have identified 6 metastasis spectra with this narrow 2.05ppm peak (assigned to the <u>H</u> in the -$CH_2$-C<u>H</u>$_2$-CH= lipid group). All show similar spectral patterns, with significant choline, high lactate and low 1.3 (-$CH_2$) and 0.9ppm (-$CH_3$) lipid peaks. The narrow 2.05ppm peak is not found in metastases spectra with very high lipids. This suggests the narrow 2.05ppm peak relates to hypoxic and/or cystic, but viable, tumour tissue prior to necrosis, and possibly more likely associated with metastases.

© 2004 Cancer Research UK

### P77

### P53 INDEPENDENT RADIATION INDUCED BYSTANDER EFFECTS INDUCED BY RADIOPHARMACEUTICALS LABELLED WITH α–, β– AND AUGER EMITTERS

M Boyd , J Dorrens , S Ross , KW Tan , N Baig , MR Zalutsky , RJ Mairs
[1] United Kingdom, CRUK Beatson Labs, Glasgow, [2] United Kingdom, Duke University, Durham, [3] United States, Glasgow School for Cancer Studies, Glasgow

Targeted radiotherapy is the selective irradiation of tumour cells by radionuclides conjugated to tumour seeking molecules. Gene therapy can expand the tumour types accessible to this type of therapy. We recently introduced the noradrenaline transporter gene (NAT) into tumour cells endowing them with the capacity for uptake of radiolabelled MIBG. Current cancer gene therapy strategies are however limited by low gene transfer efficiencies so cancer gene therapy strategies must have a component of collateral cell kill to neighbouring cells. Utilising the radiation-induced biological bystander effect (RIBBE) to its full potential could increase therapeutic efficacy. WE adapted media transfer methodology to quantify the contribution of RIBBE to cell kill in NAT transfected cells after exposure to the β–emitter [$^{131}$I]MIBG, the α-emitter [$^{211}$At]MABG or the Auger emitter [$^{123}$I]MIBG. The role of p53 in mediating RIBBE was investigated.
NAT transfected human cancer cells and their P53 null variants were irradiated using a $^{60}$Co source or by incubation with radiopharmaceutical. An amended media transfer protocol was employed to assess the magnitude of RIBBE contributing to cell kill from different targeted radiation qualities.
Dose-dependent RIBBE were identified in human cancer cell lines following γ-ray external beam radiation. RIBBE were P53 independent. Treatment with [$^{131}$I]MIBG, [$^{123}$I]MIBG and [$^{211}$At]MABG showed a substantial impact of RIBBE on cell clonogenic survival. Cell kill due to RIBBE in cells never exposed to radiation was equal to that afforded by radiopharmaceutical treatment . The level of RIBBE correlated with dose of radiopharmaceutical.
The large RIBBE observed with radiopharmaceutical treatment of NAT transfected cancer cells and the lack of requirement for functional P53, implies the feasibility of utilising this strategy to enhance the efficacy of combined gene therapy and targeted radiotherapy.

### P78

### [$^{131}$I]META-IODOBENZYLGUANIDINE AND TOPOTECAN EXPERIMENTAL COMBINATION TREATMENT OF TUMOURS EXPRESSING THE NORADRENALINE TRANSPORTER

AG McCluskey , E Cosimo , M Gaze , M Boyd , RJ Mairs
[1] United States, CRUK Beatson Labs, Glasgow, [2] United Kingdom, Middlesex Hospital, london

**Introduction:** The aim of this study was to determine the efficacy of [$^{131}$I]MIBG in combination with topotecan *in vitro* and *in vivo*.
**Results:** Two cell lines, expressing the noradrenaline transporter (NAT) were used in this study: SK-N-BE(2c) (neuroblastoma) and PN3 (NAT gene transfected glioma cell line). Three treatment schedules were assessed: topotecan administered 24h before (i), after (ii) or simultaneously with (iii) [$^{131}$I]MIBG. DNA breakage was evaluated by comet assay and cytotoxicity by clonogenic survival. Efficacy was measured by growth delay of tumor xenografts.
Supra-additivite clonogenic sterilisation was achieved by combination schedules (ii) and (iii) but not (i). The combination index values at the $IC_{50}$ level were (i) 1.379 (± 0.025) (ii) 0.761 (± 0.014) and (iii) 0.880 (± 0.016). This order of effectiveness of the sequence of treatments was reflected in their generation of long-term DNA damage: in cells assayed 24h after treatment (to allow for DNA repair), significant damage was observed following schedules (ii) and (iii) (both $p < 0.005$), but not (i) (NS).
The mean times required for a 10-fold increase in experimental tumor volume, were 18.6 days (untreated), 31.9 days ([$^{131}$I]MIBG alone), 25.3 days (TPT alone), 37.1 days (combination schedule (i)) and 49.7 days (combination schedule (ii)) whereas combination schedule (iii) cured 100% of tumors. This treatment caused no myelotoxicity, according to platelet production or stem cell clonogenic capacity.
**Conclusions:** Long-term DNA damage and supra-additive levels of toxicity to NAT expressing cells and xenografts may be achieved using a combination of TPT and [$^{131}$I]mIBG. These effects are dependent on the scheduling of the two agents.

### P79

### OESTROGEN MODULATION OF A RADIATION-INDUCED BYSTANDER EFFECT IN TARGETED BREAST CELLS

C Shao [1], KD Held [2], M Folkard [1], BD Michael [1], KM Prise [1]
[1] Gray Cancer Institute, PO Box 100, Mount Vernon Hospital, Northwood, Middlesex, HA6 2JR, United Kingdom, [2] Dept. Radiation Oncology, Cox 301, Massachusettes General Hospital, Harvard Medical School, Boston, United States

The observation of radiation-induced bystander responses where cells respond to their neighbours being irradiated may indicate an opportunity to modulate the efficacy of radiotherapy. Only limited data have been published on radiation-induced bystander effect in tumour cells and the mechanisms underpinning these responses are poorly understood. In the present study, we used the Gray Cancer Institute Charged Particle Microbeam to target precise numbers of helium ions ($^{3}He^{2+}$) to a fraction of cells within a population of human breast cancer cells of either ER-positive MCF-7 or ER-negative MDA-MB-231. It was found that when a fraction from 1% to 100% of the cells within the population was individually targeted, the yields of micronucleus (MN) of both cell lines were higher than the predicted values assuming no bystander effect. Treatment with 17β-oestradiol (E2) had no effect on the response of the MDA-MB-231 cells. However, treatment of MCF-7 cells with E2 increased both radiosensitivity and the extent of the radiation-induced bystander response. Both these increased responses were switched off when tamoxifen was present during the E2 treatment, and tamoxifen alone had no influence on the radiation response. Moreover, treatment of MCF-7 cells with L-NAME, a non-selective NOS inhibitor, decreased the yield of bystander MN, and reduced the yield of MN in the population where 100% of cells were targeted with 1 particle but not five particles. This suggests that at low doses the response to radiation is a combination of direct and bystander pathways. Unexpectedly, the L-NAME treatment did not significantly diminish the E2-enhanced radiosensitivity and bystander response. Our results indicate that more than one bystander mechanism may be functional within irradiated MCF-7 tumour cells.

### P80

### THE INFLUENCE OF THE OESTROUS CYCLE ON THE RADIATION RESPONSE OF SOLID TUMOURS

PR Swann , DJ Garvey , BA Telfer , IJ Stratford
University of Manchester, Manchester, United Kingdom

Oestrogen increases the transcription of nitric oxide synthase, thus increasing nitric oxide production, which can result in vasodilation. Fluctuating levels of oestrogen throughout the menstrual cycle has the potential to affect tumour blood flow. Variations of blood supply to a solid tumour can influence the percentage of hypoxic cells and therefore the radiation response of the tumour. The current study has evaluated the impact of the oestrous stage on the radiation response of KHT tumours in syngeneic C3H mice. The oestrous cycle consists of the stages, pro-oestrus, oestrus, metoestrus and dioestrus. Oestrogen levels peak in oestrus and fall in dioestrus. Each stage was determined by the cellular composition of vaginal smears.
Tumours were locally irradiated with 10 Gy ionising radiation at different stages of the oestrous cycle. Tumour cell survival was assessed by clonogenic assay of the excised tumour relative to untreated tumours excised at the corresponding oestrous stage. Untreated KHT tumours excised in oestrus consistently showed an increased colony forming efficiency compared to those excised at other stages of the cycle. Tumours excised immediately after irradiation in metoestrus, showed a five-fold increase in surviving fraction compared to those irradiated in oestrus.
The oestrous stage dependent effect on potentially lethal damage repair (PLDR) in KHT tumours was evaluated. The overall surviving fraction of tumours excised either immediately or 24 hours after irradiation was similar. However, the KHT tumour indicated a difference in PLDR corresponding with oestrous stage. Tumours irradiated in pro-oestrus, oestrus and dioestrus showed an increase in surviving fraction when excised 24 hours post irradiation, whereas tumours irradiated in metoestrus showed a reduction in the surviving fraction. The results presented here suggest that there are oestrous stage dependent effects that could alter the radiation response of tumours.

© 2004 Cancer Research UK

### P80:1

**RADIOPROTECTIVE GENE THERAPY FOR THE HAEMOPOETIC COMPARTMENT**

T Southgate [1], V Sheard [1], MD Milsom [1], RJ Mairs [2], M Boyd [2], LJ Fairbairn [1]
[1] United Kingdom, Paterson Institute for Cancer Research, Manchester,
[2] United Kingdom, Dept of Radiation Oncology, Cancer Research UK Beatson Laboratories, Glasgow

Overexpression of manganese superoxide dismutase (MnSOD) has been hypothesized to provide haemopoietic cells with radioprotection through the scavenging of oxygen radicals induced by ionizing radiation. For this study the human MnSOD cDNA was cloned within a retroviral construct (SFβ91) with eGFP as a marker. Retroviral producer lines were established and used to infect the human erythroleukaemic cell line, K562, and murine bone marrow.
Western blot analysis was used to confirm expression of MnSOD in target cells. Whereas control, GFP-only cells exhibited undetectable levels of MnSOD, those transduced with the SFβ91-SOD2-EGFP vector showed high levels of protein. Moreover, cells transduced with the SFβ91-SOD2-EGFP vector show MnSOD activity, whilst control cells have no detectable activity.
In transduced K562 cells, an approximately 2-fold survival advantage is sen for MnSOD-expressing cells over controls when exposed to varying doses of ionising radiation. Our findings are encouraging for the development of MnSOD gene transfer for the protection against myelotoxic side effects of radiation.

### P80:2

**H2AX NUCLEAR FOCI ARE INDUCED IN HUMAN CELLS AFTER TREATMENT WITH DNA INTERSTRAND CROSSLINKING CHEMOTHERAPEUTICS**

PH Clingen , J Wu , KM Prise , JA Hartley
[1] United States, Cancer Research UK Drug-DNA Interactions Research Group, Royal Free and University College Medical School, London,
[2] United Kingdom, Cell and Molecular Biophysics Group, Cray Cancer Institute, London

Histone H2AX is rapidly phosphorylated (γ-H2AX), forming discrete nuclear foci after exposure to ionising radiation. Using antibodies against γ-H2AX the number of nuclear foci is directly related to the number of DNA double strand breaks (dsbs). γ-H2AX has been strictly associated with this type of DNA damage and plays a critical role in the recruitment of DNA repair factors to these nuclear foci. The aim of this study was to determine if γ-H2AX foci are induced in human cells following exposure to DNA interstrand crosslinking chemotherapeutics.
Primary human fibroblasts (AGO1522B) were treated with ionising radiation (1 gray), cis-platin (5-100 μM) or nitrogen mustard (5-20 μM, 1 hour incubation at 37 °C). Cells were fixed immediately, 4, 8, 24 or 48 hours after ionising radiation or drug treatment and stained with anti phospho-H2AX and alexa fluor 488. γ-H2AX foci were detected by immunofluorescence microscopy after treatment with cis-platin and nitrogen mustard. With ionising radiation foci were evident immediately after irradiation. After 8 hours no foci were detected indicating that all the double strand breaks had been repaired. Pulse field gel electrophoresis (PFGE) and the modified comet assay were performed to follow the induction and repair of dsbs and ICLs respectively. We have previously shown that dsbs are induced in cells following nitrogen mustard but not cis-platin treatment. With both agents γ-H2AX foci formation closely follows the formation of DNA ICLs.
In conclusion, γ-H2AX foci can be observed in the absence of DNA dsbs. This approach could lead to a rapid and sensitive method to detect ICLs in clinical samples. Conventional approaches for measuring DNA ICLs, although quantitative, lack the necessary sensitivity and resolution to follow DNA damage and repair at the molecular level.

### P80:3

**H2AX PHOSPHORYLATION AS A MARKER OF DNA DAMAGE FOLLOWING DIAGNOSTIC AND THERAPEUTIC IRRADIATION *IN VIVO***

KM Rothkamm , S Balroop , F Daley
United Kingdom, Gray Cancer Institute, Northwood

Ionising radiation induces a broad spectrum of damages in our genes, of which DNA double-strand breaks (DSBs) are considered to be the most relevant lesion for cell killing, chromosome aberration formation and cancer induction. One of the earliest steps in the cellular response to DSBs is the phosphorylation of H2AX, a subclass of histone proteins that are part of the DNA-protein complex called chromatin. Using a fluorescent antibody specific for the phosphorylated form gamma-H2AX and immunofluorescence microscopy, discrete nuclear foci can be visualized at sites of DSBs. Recent cell culture studies have demonstrated that gamma-H2AX expression not only monitors the formation of DSBs but can also be exploited as a measure for the repair of radiation-induced DSBs at physiologically relevant doses. The aim of this study was to determine the usefulness of gamma-H2AX analysis for visualising DSBs after *in vivo* exposure to low or moderate doses of ionising radiation. To this end, immunofluorescence microscopy for gamma-H2AX was performed in leukocytes from blood samples of patients receiving routine CT scans to assess the DNA damage that is produced in the patient's body during these treatments. To study gamma-H2AX expression in different tissues, tissue arrays prepared from different organs of irradiated mice were immunohistochemically stained to visualise gamma-H2AX foci *in situ.* The results demonstrate that gamma-H2AX analysis enables studies of DNA damage and repair to be performed following *in vivo* exposure to diagnostic and therapeutic radiation doses.

### P81

**THE ROLE OF MYCN AMPLIFICATION AND HYPOXIA IN THE EXPRESSION OF FACILITATIVE GLUCOSE TRANSPORTERS IN NEUROBLASTOMA CELL LINES LILILINESROBLASTOMA CELL LINES**

A Lunt [1], A Evans [1], P Houghton [2], I Stratford [3], E Estlin [4], R Airley [1]
[1] Liverpool John Moores University, Liverpool, United Kingdom,
[2] St Judes Childrens Research Hospital, Memphis, United States,
[3] University of Manchester, Manchester, United Kingdom, [4] Royal Manchester Childrens Hospital, Manchester, United Kingdom

Tumour hypoxia upregulates genes such as the facilitative glucose transporters Glut-1 and Glut-3, both these genes predicting poor prognosis and increased likelihood of metastasis in a range of solid tumours. Oncogenes that code for transcription factors such as cMyc show homology with hypoxia-inducible factor HIF-1α, and therefore may cause constitutive expression of hypoxia-inducible genes. In neuroblastoma, the MYCN oncogene is associated with poor prognosis. Previously, we have shown Glut-1 to be present in a proportion of clinical samples of cases of neuroblastoma, and that this may correlate with the MYCN amplification. To investigate the influence of MYCN amplification on hypoxia-regulated genes, a study was carried out using MYCN amplified (NB-SD, NB-1691) and the single copy MYCN expressing (NB-EB) neuroblastoma cell lines. Immunohistochemistry of formalin-fixed, paraffin-embedded pellets of cells exposed to 18 hours normoxia or 0% $O_2$ was used to evaluate the presence of Glut-1, Glut-3, and Glut-4. In MYCN amplified cells, there was a low level of constitutive Glut-1 expression, which was up-regulated to moderate levels in anoxia. However, the single copy MYCN cell line (NB-EB) only expressed Glut-1 after exposure to anoxia. Glut-3 expression was only found at low levels in the NB-EB cell line, the levels being increased to moderate levels after exposure to anoxia. There was no detectable Glut-3 in the MYCN amplified cell lines. Glut-4, which is not HIF-1 regulated but is able to translocate from intracellular vesicles to the plasma membrane in hypoxic conditions, was not detectable in these cell lines. This study provides preliminary evidence that MYCN amplification and hypoxia may work together to control the expression of hypoxia-regulated genes in neuroblastoma. Further investigations will aim to clarify how MYCN amplification with or without tumour hypoxia may control the behaviour of neuroblastoma and influence treatment response and prognosis

© 2004 Cancer Research UK

### P82

### IS HYPOXIA A THERAPEUTIC TARGET IN HAEMATOLOGICAL MALIGNANCIES? GLUT-1 AND LDH EXPRESSION IN ACUTE LYMPHOBLASTIC LEUKAEMIA

K Santos [1], A Evans [1], A Kelsey [2], R Wynn [2], E Estlin [2], R Airley [1]
[1] Liverpool John Moores University, Liverpool, United Kingdom,
[2] Royal Manchester Childrens Hospital, Manchester, United Kingdom

Hypoxia traditionally occurs within solid tumours due to the high rate of cell proliferation and poor blood supply. It is associated with radio and chemoresistance, poor prognosis and metastasis. Hypoxia is not associated with leukaemia since it does not form solid tumours; however, these cells originate from within the bone marrow, in which a low oxygen tension is possible due to the high proliferation of these lymphoid cells. Therefore, hypoxia in the bone marrow may influence the downstream behaviour of leukaemic cells. We are therefore carrying out studies to investigate the expression of the hypoxia-regulated genes Glut-1 and lactate dehydrogenase (LDH) in acute lymphoblastic leukaemia (ALL). We have previously validated Glut-1 as a marker of hypoxia and a marker of prognosis in advanced carcinoma of the cervix, oral squaemous carcinoma and colorectal tumours. Raised serum LDH is a poor prognostic indicator in ALL. Further, tissue LDH-5 predicts poor prognosis in non-small cell lung cancer. LDH-1 is found in all cells, malignant and normal, and due to its low catalytic activity promotes the insertion of pyruvate into the Kreb's Cycle to facilitate aerobic oxidative phosphorylation. LDH-5, however, has high catalytic activity. Therefore, where oxygen is lacking, anaerobic glycolysis may be accompanied by an increased rate of conversion of pyruvate to lactate by LDH-5. So far, the pattern of expression of Glut-1 and LDH isoforms in haematological malignancies is poorly understood. To address this, Glut-1, LDH-1 and LDH-5 expression is currently being investigated in clinical samples and formalin-fixed, paraffin-embedded cell pellets of MOLT-4 (T-cell) and NALM-6 (B-cell) ALL cell lines that have been exposed to anoxia. In a series of trephine biopsies taken from children with ALL, Glut-1 protein was detected in lymphoblasts of 3/10 cases. By characterising the pattern of expression of these hypoxia-dependent genes we may be able to predict the influence of hypoxia in haematological malignancies, as well as the relative levels of acute and chronic hypoxia existing in clinical samples.

### P83

### HYPOXIA-REGULATED PROTEINS (HRPS) AND MATRIX METALLOPROTEINASES (MMPS) IN GASTRIC CANCER: IMPACT ON MALIGNANT PROGRESSION

S.P. Osinsky , L.N. Bubnovskaya , I.I. Ganusevich , A.V. Kovelskaya, O.V. Yurchenko , N.V. Valkovskaya , D.S. Osinsky , L.D. Gumenyuk , V.A. Chorny
Inst. exp. Pathol. Oncol. Radiobiol., Natl Acad, Sci. of Ukraine, Kiev, Ukraine

**Aim:** to evaluate the interrelationship between hypoxia level, expression of HRPs and activity of MMPs in tumor tissue and assess the impact of these parameters on malignant progression.
**Patients&Methods:** 43 gastric cancer patients who underwent primary surgery were included in the study. All patients gave written consent before samples were obtained. Activity of MMPs (MMP-2 and -9) in tumor was determined with biochemical methods; hypoxia within tissue was evaluated using $^{31}$P NMR spectroscopy, expressions of hypoxia-inducible factor-1alpha (HIF-1alpha) and CD34 (microvessel density) in tissue were assessed using immunohistochemistry. All human tumor indices were measured immediately after surgery. Lewis lung carcinoma (3LL) was investigated in model experiments too.
**Results:** The positive correlation between hypoxia level and MMPs activity in primary tumor of 3LL tumor-bearing mice was observed. Both indices had positively impact on lung metastases. The hypoxia level assessed with $^{31}$P NMR correlated with HIF-1alpha expression in human gastric cancer (HGC). The direct correlation between hypoxia level, tumor stage and CD34 expression was determined in HGC. Tumor MMPs activity was significantly higher than that in the surrounding normal mucosa. It was not observed the correlation between MMPs activity and CD34 expression in tumor. The significant correlation between tumor hypoxia and MMPs activity was not determined. It may be suggested that MMPs activity is not directly affected by hypoxia in HGC. It was not observed the correlation between MMPs activity and tumor stage. Hypoxia level and MMPs activity positively correlated with disease-free termin and metastases.
**Conclusion:** Hypoxia positively impacts on malignant progression. HIF-1alpha and CD34 expressions and MMPs activity in tumor tissue may be exploited as an prognostic markers for clinical outcome.
Acknowledgement: the study was supported by SNFS (SCOPES).

### P84

### INVESTIGATION OF THE ROLE OF GLUCOSE TRANSPORTER-1, AS A MARKER FOR TUMOUR HYPOXIA, IN NEO-ADJUVANT RADIOTHERAPY OF RECTAL CANCER

NP Yeomans , MC Bibby , RM Phillips , JP Griffith , JB Davies
United Kingdom, The University of Bradford, Bradford

Neo-adjuvant short course, high dose radiotherapy is advocated for local control, reduced recurrence and improving survival in certain rectal adenocarcinomas, when compared to surgery alone. The representative volume of hypoxic cells in colorectal cancers is reported to vary from as little as 1% to over 80% which leads to an adaptation towards anaerobic metabolism, via glycolysis, and tumour radio-resistance. Glucose transporter-1 (Glut-1) is the main transmembrane glucose transporter up-regulated in many human cancers and is one of many transcription factors of hypoxia inducible factor-1 (HIF-1).
In this preliminary series of 20 patients receiving neo-adjuvant radiotherapy for rectal cancer there was no significant difference in the tumour hypoxic fraction before and after radiotherapy but with a broad range of 0.5% to 28% (mean 6%) as indicated by Glut-1 immunohistochemical tissue section staining.
There was no significant difference in hypoxic fraction when compared to Dukes' or T stage but those deemed to have a good response to radiotherapy did have a lower hypoxic fraction when compared to the other rectal tumours.

### P85

### HYPOXIA DRIVEN TNF-$\alpha$ GENE THERAPY

EJ Garside , R Cowen , K Williams , N Chadderton , IJ Stratford
Experimental Oncology, Department of Pharmacy and Pharmaceutical Sciences, University of Manchester, Manchester, United Kingdom

TNF$\alpha$ has strong anticancer activity but systemic toxicity limits its clinical use. Hypoxia responsive elements (HREs) can be used to drive gene expression in hypoxia. Salloum et al (2003) used the erythropoietin HRE (Epo) with Egr1 to drive TNF$\alpha$. In our experience this promoter gives high aerobic expression that prevents its systemic use. Here we present a hypoxia responsive adenovector Ad LDH TNF that carries a synthetic HRE promoter derived from lactate dehydrogenase (LDH). We have previously demonstrated the utility of this promoter in a hypoxia driven GDEPT strategy (Cowen et al 2004, Cancer Res.). In transient plasmid transfection experiments we have compared the LDH and Epo-Egr1 promoters driving expression of luciferase in aerobic and hypoxic tumour cells *in vitro*. The LDH promoter silences reporter gene expression in aerobic tumour cells compared to EpoEgr1 [HCT116 (colon carcinoma) 6 fold ↓ and HT1080 (fibrosarcoma) 3 fold ↓ ]. Unlike other tumour specific promoters absolute levels of expression are not compromised once the LDH promoter is stimulated by $O_2$ tensions at or below 1% $O_2$. In comparison to the Epo-Egr1 promoter the strength of expression is equivalent in the HT1080 cells and 50% higher in the HCT116 cells. When we replace the luciferase gene with the cDNA for human TNF$\alpha$ we are able to confer tight hypoxic control. In supernatants taken from aerobic HCT116 and HT1080 cells transfected with pLDH TNF$\alpha$ we are unable to detect TNF$\alpha$. However, cells stimulated by hypoxia (0.001% $O_2$) or 1% $O_2$ secrete high levels of the cytokine (HT1080 ~ 3618 and 3215 pg/ml; HCT116 ~ 5395 and 6170 pg/ml) In contrast TNF$\alpha$ secretion from Epo-Egr1 was independent of $O_2$ concentration (HT1080 ~ 1306, 6439, 4794 pg/ml and HCT116 ~ 5498, 7712, 8362 pg/ml in air, hypoxia and 1% $O_2$). We are now generating adenovectors to deliver luciferase or TNF$\alpha$ with expression driven by LDH or Epo-Egr-1. Using these viral vectors we will undertake *in vivo* studies and envisage that aerobic silencing will translate into tumour specific expression when delivered with minimal unwanted expression in normal tissues.

### P86

**SCREENING OF POTENTIAL HIF-1 MODULATOR DRUGS IN HCT116 CELL LINE : MORE TOXICITY THAN SPECIFIC INHIBITION OF HIF-1**

C Debray , KJ Williams , R Cowen , IJ Stratford
Experimental Oncology, School of Pharmacy and Pharmaceutical Sciences, University of Manchester, Manchester, United Kingdom

Hypoxia-inducible factor 1 (HIF-1) is a key transcription factor in the regulation of the response to hypoxia. Its upregulation in many solid tumours and its involvement in tumour progression make it a potential target for anticancer therapy. It binds to hypoxia response elements (HRE) present in the promoters of genes controlling angiogenesis, cell proliferation and cell survival. Recent works suggest that HIF-1 can be regulated by the PI3K/Akt and the MAPK pathways and by the association with the molecular chaperone heat shock protein 90 (Hsp90). We tested a range of drugs that have been proposed to modulate HIF-1 driven gene expression. These include drugs targeting the PI3K pathway (LY294002, Wortmannin, Rapamycin), the MAPK pathway (PD98059), the Hsp90-specific inhibitor geldanamycin and YC-1. We have developed a human colon adenocarcinoma HCT116 stable cell line engineered to encode for an HRE driven firefly luciferase gene and importantly a constitutive promoter driven renilla luciferase cassette as an internal control. We quantified firefly and renilla luciferase in the presence of each drug with and without the hypoxia mimetic $CoCl_2$. The toxicity of these molecules was evaluated by MTT survival assays. At drug concentrations shown to modulate HIF-1 in the literature in our screen most of the drugs had only a toxic effect and no specific inhibition of HIF-1 activity. Geldanamycin was toxic at 5-10 nM and did not inhibit HIF-1 at non toxic concentrations. LY294002 and Rapamycin showed neither toxicity nor inhibitory effect and Wortmannin and YC-1 modestly decreased HIF-1 activity in $CoCl_2$ conditions. However, PD98059 showed a specific inhibition of HIF-1 activated firefly luciferase expression that was greater in $CoCl_2$ condition. The exact mechanisms of this inhibition are currently under investigation.

### P87

**THE ROLE OF HYPOXIA AND THE TRANSCRIPTION FACTOR HIF- 1 IN THE DEVELOPMENT OF METASTASIS**

LK Whitney , KJ Williams , IJ Stratford , SJ Lunt , L Brown
Experimental Oncology, Department of Pharmacy and Pharmaceutical Sciences, University of Manchester, Manchester, United Kingdom

Tumour hypoxia and the hypoxic/HIF-1 signalling pathway have been linked to an increased incidence of metastases. To investigate the role of HIF-1 in metastatic potential we are utilising the syngeneic murine melanoma B16 F1/C57BL6 metastatic model that reproducibly forms metastases at various sites following irradiation of subcutaneous primary tumours. We have transiently transfected B16 F1 cells *in vitro* with a hypoxia responsive luciferase reporter plasmid to confirm that the cell line has an intact hypoxic response and witnessed up to a 500-fold induction in luciferase following a hypoxic (0.001% $O_2$) stimulus. This paved the way for the generation of a HIF-1 responsive metastatic reporter cell line. The cell line was generated by stable integration of a lacZ expression cassette whose promoter contains synthetic hypoxia responsive enhancer elements from murine phosphoglycerate kinase (PGK HRE). The process of clonal selection had not diminished the metastatic potential, and both Z16 primary and metastatic tumours stain positive for beta galactosidase expression that co-localises with the hypoxic marker pimonidazole. We are currently generating a stable cell line that constitutively expresses beta galactosidase by inserting the lacZ cDNA into pEF IRES puro an optimised vector for stable cell line generation. Having established the model to investigate the HIF-1 dependency of metastases formation we have created a dominant negative form of HIF-1 alpha that is constitutively expressed and lacks the oxygen degradation and transactivation domains. Transient co-transfections in B16 F1 cells with the dominant negative and a PGK HRE luciferase reporter plasmid demonstrates functionality of the dominant negative inhibiting luciferase expression in hypoxic cells down to basal aerobic levels. Stable cell lines are now being generated by integration of the dominant negative cassette into the Z16 cells.

### P88

**MANIPULATION OF TUMOUR HYPOXIA USING TNF ALPHA GENE THERAPY TO POTENTIATE BIOREDUCTIVE DRUGS AND/OR HYPOXIA DRIVEN GENE THERAPY**

RL Cowen , E Garside , KJ Williams , FC Sheppard , BA Telfer , M Jaffar , IJ Stratford
United Kingdom, University of Manchester, Manchester

The refractive nature of hypoxic tumour cells to current anti-cancer treatments makes them an attractive target for novel gene therapies. Our group's approach has been to hijack the hypoxic/HIF pathway by using it as a trigger for tumour selective gene therapy. HIF initiates transcription via hypoxia responsive enhancer elements (HREs) therefore by engineering synthetic HREs into therapeutic cassettes we have been able to confer hypoxic tumour specificity to both adenoviral delivered enzyme/prodrug (P450 reductase/Tirapazamine) and suicide (TNF alpha) gene therapy approaches.

The tumour vascular system is responsible for the oxygenation of cells therefore by targeting endothelial cells we can manipulate tumour hypoxia. Interestingly, the tumour endothelium is the proposed target for TNF alpha. Work by Edwards et al., (1991) demonstrated that recombinant TNF alpha could induce rapid haemorrhagic necrosis in murine KHT tumours resulting in tumour hypoxia equivalent to 100% radiobiological hypoxia. This effect was transient occurring within 1h of administration and lasting for 16h. Therefore we hypothesise that by using TNF alpha gene therapy we can potentially increase the efficacy of our hypoxia driven gene therapy approaches.

We are currently determining the influence of the TNF alpha expressing adenovectors upon endothelial cells and tumour hypoxia in vivo. We are also investigating their ability to potentiate bioreductive drugs such as TPZ. Further we envisage a greater than additive effect from combining our P450 reductase/Tirapazamine and TNF alpha gene therapy approaches.

### P89

**TARGETING HIF-1 TO REVERSE TUMOUR CHEMO-RESISTANCE**

A Eustace , KJ Williams , RL Cowen , L Brown , BA Telfer , IJ Stratford
United Kingdom, Experimental Oncology, Department of Pharmacy and Pharmaceutical Sciences, University of Manchester, Manchester

Hypoxic tumour cells are resistant to many forms of chemotherapy agents. We are investigating the mechanisms involved and whether targeting HIF-1 using gene therapy will reverse this resistance. We have determined by MTT assay that hypoxic human HT1080 (fibrosarcoma) and HCT116 (colon carcinoma) tumour cells are more resistant to etoposide than aerobic tumour cells (2-4 fold). This hypoxic resistance was also observed in wild-type Hepa-1 (murine hepatoma) cells. However, it was not seen in Hepa-1 HIF-1 beta deficient cells suggesting a HIF-1 dependency for resistance. To target HIF-1 we have created a dominant negative form of HIF-1 alpha (HIF-1 alpha no TAD) that is constitutively expressed and lacks the transactivation domains. Transient co-transfection experiments in HT1080 and HCT116 cells with the HIF-1 alpha no TAD and the hypoxia responsive (PGK HRE) luciferase reporter expression plasmids demonstrate functionality of the dominant negative. We have also created a HIF-1 alpha no TAD/GFP fusion and have demonstrated that GFP fluorescence and inhibition of hypoxia driven luciferase expression is not compromised. GFP tagging has also confirmed that the dominant negative is nuclear localised in aerobic and hypoxic cells. We have inserted this fused cDNA into pEF IRES puro an optimised vector for stable cell line generation. We have also engineered a replication defective adenovector Ad HIF-1 alpha no TAD. Using the virus we can re-capitulate the plasmid experiments inhibiting hypoxia driven luciferase expression in a viral dose dependent manner. Further, we show that we can reverse hypoxia mediated etoposide resistance in HT1080 and HCT116 by infection with Ad HIF-1 alpha no TAD two days prior to drug exposure. We aim to extend these observations to other agents and investigate whether targeting HIF-1 transactivation using Ad HIF-1 alpha gene therapy can modify drug response *in vivo*.

© 2004 Cancer Research UK

### P90

### P43/EMAP-II EXPRESSION IN COLORECTAL CANCER IS ASSOCIATED WITH HYPOXIA AND ENHANCED LYMPHOCYTE INFILTRATION

M Saber , P Symonds , JC Murray
Wolfson Digestive Diseases Centre, Queen's Medical Centre, Nottingham University, Nottingham, United Kingdom

**AIMS**: P43/Endothelial monocyte–activating polypeptide-II (p43/EMAP-II) is a proinflammatory cytokine and a chemoattractant for mononuclear phagocytes and polymorphonuclear leucocytes, found in culture supernatants of many tumour cell lines. We recently demonstrated that p43/EMAP-II induces apoptosis in mitogen-stimulated lymphocytes, and suggested that it may be a constituent of a novel immune evasion mechanism employed by tumour cells (1). Furthermore p43/EMAP-II release is enhanced by hypoxia (2). Our study has examined the association between p43/EMAP-II expression and hypoxia in colorectal cancer (CRC), and also the association between p43/EMAP-II and tumour cell apoptosis.
**METHODS**: Formalin-fixed, paraffin-embedded archival tissue samples from 72 patients diagnosed with colorectal tumours was used in immuno-histochemical studies. Antibodies against p43/EMAP-II, carbonic anhydrase (CA IX) as a surrogate marker of hypoxia, and CD3 to identify lymphocytes, were used. Areas of p43/EMAP-II and CA IX staining were quantified using computer-aided image analysis.
**RESULTS**: P43/EMAP-II expression was correlated with CA IX expression in CRC ($p$=0.03). Patients with high p43/EMAP-II expression seemed to do better than those with low, and the reverse was true for CA IX. There was also a positive correlation between p43/EMAP-II and the lymphocyte counts in CRC ($p$=0.02), as well as between CA IX and lymphocyte counts. The presence of CD3+ve cells was a good prognostic indicator in terms of overall survival.
**CONCLUSIONS**: P43/EMAP-II expression is associated with hypoxia in colorectal cancer, and with high lymphocyte counts. We are currently determining the relationship between p43/EMAP-II expression and the functional state of the lymphocyte population.

(1) Murray *et al*. (2004). *J Immunol* **172**: 274.
(2) Barnett *et al.* (2000). *Cancer Research* **60**: 2850.

© 2004 Cancer Research UK

